# Imaging tumor lactate is feasible for identifying intermediate-risk prostate cancer patients with postsurgical biochemical recurrence

**DOI:** 10.1073/pnas.2312261120

**Published:** 2023-11-27

**Authors:** Nikita Sushentsev, Gregory Hamm, Jack Richings, Mary A. McLean, Ines Horvat Menih, Vinay Ayyappan, Ian G. Mills, Anne Y. Warren, Vincent J. Gnanapragasam, Simon T. Barry, Richard J. A. Goodwin, Ferdia A. Gallagher, Tristan Barrett

**Affiliations:** ^a^Department of Radiology, Addenbrooke’s Hospital and University of Cambridge, Cambridge Biomedical Campus, CB2 0QQ Cambridge, United Kingdom; ^b^Imaging and Data Analytics, Clinical Pharmacology and Safety Sciences, Research & Development, AstraZeneca, Cambridge CB2 0AA, United Kingdom; ^c^Patrick G. Johnston Centre for Cancer Research, Genito-Urinary and Prostate Focus Group, Queen’s University Belfast, Belfast BT9 7AE, United Kingdom; ^d^Nuffield Department of Surgical Sciences, University of Oxford, John Radcliffe Hospital, Oxford OX3 7DQ, United Kingdom; ^e^Centre for Cancer Biomarkers, Department of Clinical Science, University of Bergen, Bergen 5021, Norway; ^f^Department of Pathology, Cambridge University Hospitals National Health Service Foundation Trust, CB2 0QQ Cambridge, United Kingdom; ^g^Department of Urology, Cambridge University Hospitals National Health Service Foundation Trust, Cambridge CB2 0QQ, United Kingdom; ^h^Cambridge Urology Translational Research and Clinical Trials Office, Cambridge Biomedical Campus, Addenbrooke’s Hospital, Cambridge CB2 0QQ, United Kingdom; ^i^Bioscience, Discovery, Oncology Research & Development, AstraZeneca, Cambridge CB20AA, United Kingdom

**Keywords:** prostate cancer, cancer metabolism, MRI

## Abstract

While radical prostatectomy remains the mainstay of prostate cancer (PCa) treatment, 20 to 40% of patients develop postsurgical biochemical recurrence (BCR). A particularly challenging clinical cohort includes patients with intermediate-risk disease whose risk stratification would benefit from advanced approaches that complement standard-of-care diagnostic tools. Here, we show that imaging tumor lactate using hyperpolarized ^13^C MRI and spatial metabolomics identifies BCR-positive patients in two prospective intermediate-risk surgical cohorts. Supported by spatially resolved tissue analysis of established glycolytic biomarkers, this study provides the rationale for multicenter trials of tumor metabolic imaging as an auxiliary tool to support PCa treatment decision-making.

Radical prostatectomy (RP) is a definitive treatment option for patients with clinically localized prostate cancer (PCa), but unfortunately, 20 to 40% will develop postsurgical biochemical recurrence (BCR) (1). BCR prediction is particularly challenging in patients with intermediate-risk PCa who often face uncertainty when deciding on the best treatment (2). Current tools for preoperative BCR risk assessment only include standard clinical parameters, while recently developed models incorporating multiparametric MRI (mpMRI) and targeted biopsy data are yet to enter clinical guidelines (3). The performance of mpMRI in this patient group may be further improved through the development of novel imaging techniques such as hyperpolarized [1-^13^C]pyruvate MRI (HP-^13^C-MRI), which probes tumor [1-^13^C]lactate labeling as a feature of glycolytic metabolism (4), a phenotype independently associated with postsurgical BCR (5–7). While preoperative [1-^13^C]lactate labeling is capable of both intergrade (8) and intragrade (9) tumor differentiation, it has not yet been linked to surgical outcomes, which limits our understanding of the true potential of HP-^13^C-MRI to tease out aggressive lesions and influence clinical decision-making in the pretreatment setting. This prospective study focused on resolving this by correlating tumor lactate imaging using HP-^13^C-MRI and spatial metabolomics with surgical outcomes in two prospective cohorts of patients with intermediate-risk PCa.

## Results

**Fig. 1.**
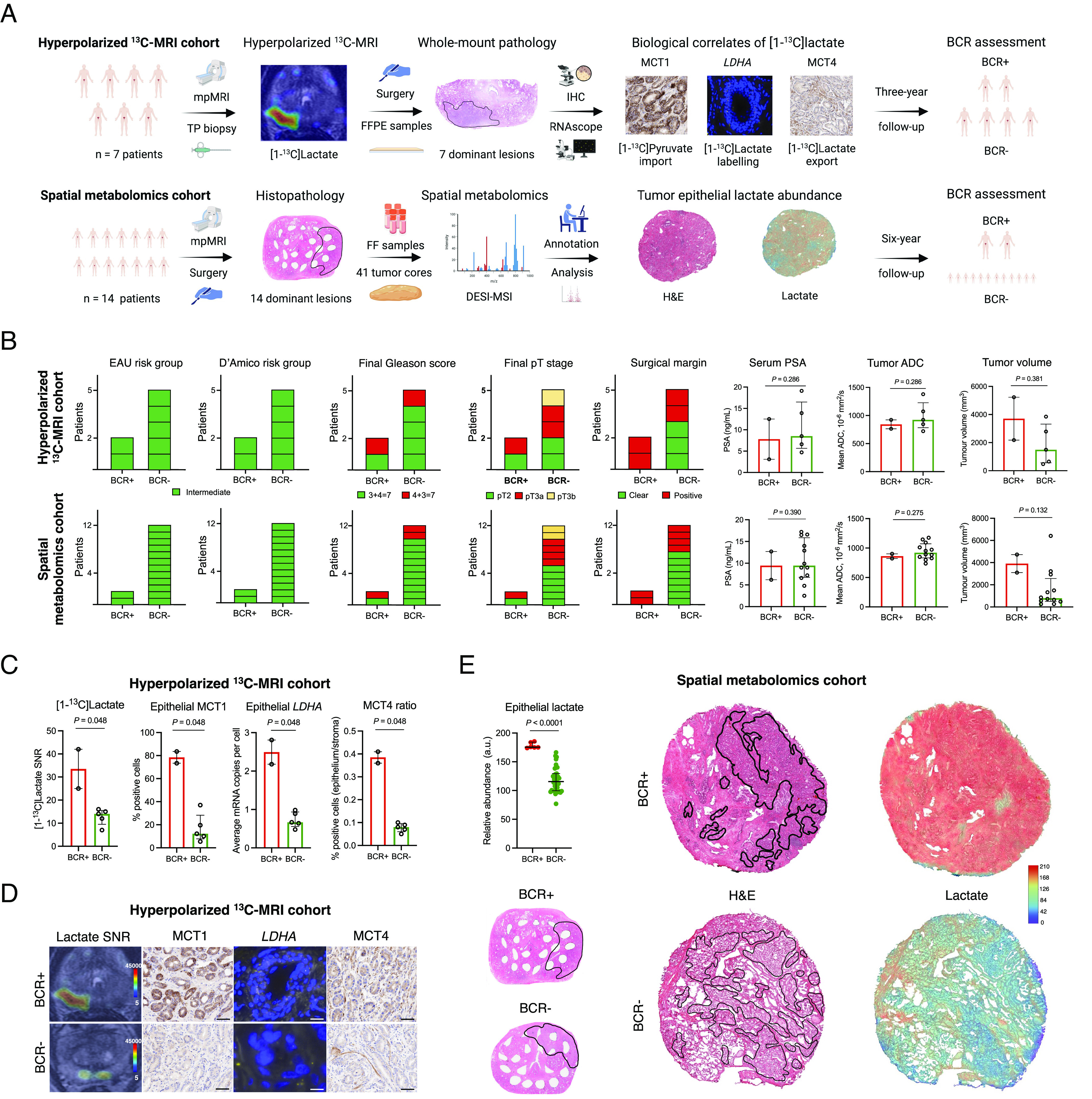
Clinical lactate imaging for BCR prediction in intermediate-risk PCa patients. (*A*) Study flowchart presenting the HP-^13^C-MRI (*Top*) and spatial metabolomics (*Bottom*) patient cohorts; this panel was created using BioRender. (*B*) Clinicopathological characteristics of BCR-positive and BCR-negative patients from the HP-^13^C-MRI (*Top*, *N* = 7) and spatial metabolomics (*Bottom*, *N* = 14) patient cohorts. (*C*) Comparison of imaging and tissue-based metabolic characteristics of BCR-positive (*N* = 2) and BCR-negative (*N* = 5) lesions from the HP-^13^C-MRI cohort. (*D*) Representative images from the HP-^13^C-MRI cohort. Scale bars denote 5 to 50 μm. (*E*) DESI-MSI-derived tumor epithelial lactate comparison between BCR-positive (*N* = 2) and BCR-negative (*N* = 12) lesions from the spatial metabolomics cohort.

The primary cohort included seven newly diagnosed PCa patients who underwent HP-^13^C-MRI prior to RP (9) and were monitored in our center for a minimum of 3 y (range, 36-58 mo) (Fig. 1 *A*, *Top*). Preoperatively, all patients had intermediate risk of BCR development according to the European Association of Urology and D’Amico risk groups (Fig. 1 *A*, *Top*). The same was true for the matched secondary cohort of 14 PCa patients who were followed up for a minimum of 6 y after RP (range, 76 to 90 mo) with a total of 41 tumor cores sampled for the spatial metabolomics analysis (Fig. 1 *A* and *B*, *Bottom*). Two patients in each cohort developed BCR at 16 and 22 mo (HP-^13^C-MRI cohort), as well as 11 and 18 mo (spatial metabolomics cohort) after surgery, respectively. In both cohorts, one BCR-positive patient had pT3a disease, and both BCR-positive patients had positive surgical margins; importantly, these two adverse histopathological characteristics were also noted in some BCR-negative patients (Fig. 1*B* and Table 1). In both cohorts, mpMRI-derived tumor apparent diffusion coefficient (ADC) values and tumor volumes, as well as serum prostate-specific antigen (PSA) measurements, were similar between BCR-negative and BCR-positive patients (Fig. 1*B* and Table 1).

**Table 1.** Tumor imaging and biological features in patients with and without biochemical recurrence following radical prostatectomy

Parameter	Tumors in BCR-positive patients	Tumors in BCR-negative patients	*P*
Hyperpolarized ^13^C-MRI cohort			
Tumor ADC, 10^−6^ mm^2^/s	843 (763 to 922)	923 (783 to 1,227)	0.286
Tumor volume, mm^3^	3,704 (2,179 to 5,229)	1,492 (563 to 3,318)	0.381
Serum PSA, ng/mL	7.8 (3.1 to 12.5)	8.5 (5.7 to 16.5)	0.286
Lactate signal-to-noise ratio	34 (25 to 42)	14 (10 to 15)	0.048
Epithelial *LDHA*, average mRNA copies per cell	2.50 (2.18 to 2.81)	0.67 (0.56 to 0.94)	0.048
Epithelial MCT1, % positive cells	78.55 (73.36 to 83.51)	12.21 (7.81 to 28.30)	0.048
Epithelium-to-stroma MCT4 ratio, % positive cells	0.39 (0.36 to 0.41)	0.08 (0.06 to 0.10)	0.048
Spatial metabolomics cohort			
Tumor ADC, 10^−6^ mm^2^/s	862 (822 to 901)	922 (833 to 1,073)	0.132
Tumor volume, mm^3^	3,916 (3,103 to 4,728)	791 (504 to 2,567)	0.132
Serum PSA, ng/mL	9.5 (6.2 to 12.7)	9.5 (6.8 to 15.9)	0.390
Tumor epithelial lactate abundance, a.u.	175 (84 to 175)	116 (100 to 129)	<0.0001

The data are presented as median (interquartile range). *P* were derived using a one-tailed Mann–Whitney *U* test.

Notably, in the HP-^13^C-MRI cohort, BCR-positive patients showed significantly elevated preoperative tumor [1-^13^C]lactate labeling (Fig. 1 *C* and *D* and Table 1). At the tissue level, tumors in BCR-positive patients also demonstrated a significant increase in the epithelial immunoexpression of monocarboxylate transporter 1 (MCT1) (Fig. 1 *C* and *D* and Table 1), a key intracellular importer of [1-^13^C]pyruvate (8, 10). In addition, BCR-positive lesions showed the highest epithelial mRNA expression of lactate dehydrogenase A (*LDHA*) (Fig. 1 *C* and *D* and Table 1), an enzyme catalyzing the [1-^13^C]pyruvate-to-[1-^13^C]lactate conversion. Finally, BCR-positive lesions also showed the highest epithelium-to-stroma monocarboxylate transporter 4 (MCT4) ratio (Fig. 1 *C* and *D* and Table 1), an independent BCR predictor (7) and tissue correlate of [1-^13^C]lactate labeling (9). Importantly, in the spatial metabolomics cohort, tumor epithelial lactate measured using desorption electrospray ionization mass spectrometry imaging (DESI-MSI) was also significantly increased in samples obtained from BCR-positive patients (Fig. 1*E* and Table 1).

## Discussion

This prospective study suggests the feasibility of using both invasive (DESI-MSI) and noninvasive (HP-^13^C-MRI) novel clinical metabolic imaging tools in intermediate-risk PCa patients to identify men harboring metabolically active lesions at increased risk of surgical failure. In addition to reporting imaging findings, we attempted to mechanistically explain our observations through spatially resolved tissue analysis of established glycolytic biomarkers, corroborated by the direct epithelial lactate readout using spatial metabolomics. Future work will involve multi-institutional validation of our preliminary findings in larger cohorts to prospectively determine the clinical impact of metabolic imaging on PCa care.

## Materials and Methods

Prior to surgery, all patients from the HP-^13^C-MRI cohort provided written informed consent for participation in the MISSION-Prostate prospective study that was approved by the institutional review board (National Research Ethics Service Committee East of England, Cambridge South, Research Ethics Committee number 16/EE/0205) and involved presurgical HP-^13^C-MRI acquisition, biological analysis of surgical samples, and postsurgical follow-up reported in this study. The DESI-MSI analysis was conducted under an Institutional Review Board-approved prospective national study (DIAMOND, National Research Ethics Service Committee East of England, Cambridge South, Research Ethics Committee number 03/018), which involved prospective collection of fresh frozen radical prostatectomy samples from patients who provided written informed consent for their subsequent retrieval and analysis under the study protocol. Detailed imaging and tissue analysis protocols for the HP-^13^C-MRI cohort are provided in the original cohort description (9). Spatial metabolomics analysis in the secondary cohort was performed by means of desorption electrospray ionization mass spectrometry imaging, with a detailed protocol provided in *SI Appendix*.

## Supplementary Material

Appendix 01 (PDF)

## Data Availability

Study dataset have been deposited in Mendeley Data (https://doi.org/10.17632/cxpc52bwhn.1) (11).
