# Supplementary material for: Imaging tumor lactate is feasible for identifying intermediate-risk prostate cancer patients with postsurgical biochemical recurrence
Source: Proc Natl Acad Sci U S A. 2023 Nov 27;120(49):e2312261120. doi: 10.1073/pnas.2312261120 (PMC10710070; doi:10.1073/pnas.2312261120)
Supplement: Supplementary file 1 — Appendix 01 (PDF) [file pnas.2312261120.sapp.pdf]

PNAS

Supporting Information for

Imaging tumor lactate is feasible for identifying intermediate-risk prostate cancer patients with post-surgical biochemical recurrence

Nikita Sushentsev[1†*], Gregory Hamm[2†], Jack Richings[2], Mary A. McLean[1], Ines Horvat Menih[1], Vinay Ayyappan[1], Ian G. Mills[3,4,5,6], Anne Y. Warren[7], Vincent J. Gnanapragasam[8,9], Simon T. Barry[10††], Richard J. A. Goodwin[2††], Ferdia A. Gallagher[1††], Tristan Barrett[1††]

[1] Department of Radiology, Addenbrooke's Hospital and University of Cambridge, Box 218, Cambridge Biomedical Campus, CB2 0QQ, Cambridge, United Kingdom

[2] Imaging and Data Analytics, Clinical Pharmacology & Safety Sciences, R&D, AstraZeneca, Cambridge, UK

[3] Patrick G Johnston Centre for Cancer Research, Queen's University Belfast, Belfast, UK

[4] Nuffield Department of Surgical Sciences, University of Oxford, John Radcliffe Hospital, Oxford, UK

[5] Centre for Cancer Biomarkers, University of Bergen, Bergen, Norway

[6] Department of Clinical Science, University of Bergen, Bergen, Norway

[7] Department of Pathology, Cambridge University Hospitals NHS Foundation Trust, Hills Road, CB2 0QQ, Cambridge, United Kingdom

[8] Department of Urology, Cambridge University Hospitals NHS Foundation Trust, Cambridge, UK

[9] Cambridge Urology Translational Research and Clinical Trials Office, Cambridge Biomedical Campus, Addenbrooke's Hospital, Cambridge, UK

[10] Bioscience, Discovery, Oncology R&D, AstraZeneca, Cambridge, UK

* Dr Nikita Sushentsev

Email: ns784@medcshl.cam.ac.uk

† These authors contributed equally to this project.

†† These authors jointly supervised this work.

Author Contributions: N.S., G.H., F.A.G., and T.B. designed research; N.S. G.H., J.R., M.A.M., I.H.M., and A.Y.W. performed research; N.S., G.H., J.R., M.A.M., V.A., and I.G.H. analyzed data; N.S., G.H., F.A.G., and T.B. wrote the manuscript; V.J.G., S.T.B., R.J.A.G., F.A.G. and T.B. critically revised the manuscript, provided materials for research, and supervised its conduct.

Competing Interest Statement: G.H., J.R., S.T.B., and R.J.A.G. are AstraZeneca employees. F.A.G. has research support from GE Healthcare, grants from GlaxoSmithKline, and has consulted for AstraZeneca on behalf of the University of Cambridge. The remaining authors declare no competing interests.

Classification: Biological Sciences; Medical Sciences.

Keywords: prostate cancer, cancer metabolism, magnetic resonance imaging,

This file includes: SI Appendix

SI Appendix

Patients

The primary cohort described in this work was enrolled in the MISSION-Prostate (Molecular Imaging and Spectroscopy with Stable Isotopes in Oncology and Neurology—Imaging metabolism in prostate) prospective study that was approved by the institutional review board (National Research Ethics Service Committee East of England, Cambridge South, Research Ethics Committee number 16/EE/0205). The study enrolled seven treatment-naïve PCa patients who underwent HP-^{13}C-MRI prior to RP and were monitored in our centre for a minimum of three years. The inclusion criteria, patient characteristics, and methodology have been detailed previously (1), with tumour-derived imaging, IHC, and spatial transcriptomics data derived using the total carbon SNR-guided approach described in our prior work (2).

For the secondary analysis, we retrieved fresh frozen radical prostatectomy samples originally obtained in our centre as part of a prospective national study (DIAMOND, National Research Ethics Service Committee East of England, Cambridge South, Research Ethics Committee number 03/018), with informed consent obtained from all patients. Patients whose cores were retrieved as part of this study were matched to the primary MISSION-Prostate cohort by the key clinicopathological characteristics presented in **Fig. 1B** of the main text.

Spatial metabolomics

As part of the prospective DIAMOND study, RP specimens were transferred on ice to the laboratory within 30 min of surgical resection, with multiple punch biopsies removed using a standard 4-6 mm skin punch as described previously (3). The sites of the punched cores were marked on a map of the organ, from which cores including tumour tissue (n = 41) were selected for this study by an experienced genitourinary pathologist (A.Y.W.) who handled sample preparation.

For the MSI experiment, the punched cores were embedded and prepared according to a previously reported sample preparation workflow (4). Briefly, the punched cores were co-embedded in a (Hydroxypropyl)-methylcellulose (HPMC) + Polyvinylpyrrolidone (PVP) hydrogel to enable time-efficient sectioning under comparable conditions for all specimens analysed in one experiment. A total of 41 punched cores were placed upright in peel-a-way moulds (Thermo Scientific, Waltham, MA, USA) pre-filled with ice-cold embedding medium. Snap freezing of the filled mould was performed in dry ice-chilled isopropanol followed by a wash in dry ice chilled iso-pentane to wash off the excess of isopropanol. The frozen moulds were kept on dry ice to allow evaporation of the adherent iso-pentane before sectioning. The resulting block was sectioned to 10μm thickness using a CM3050 cryo-microtome (Leica Biosystems, Nussloch, Germany) and thaw-mounted onto Superfrost slides (Fisher Scientific, Loughborough, UK) for DESI MSI and H&E examination. Two levels were taken from the block separated by 250 μm. Tissue section slides were vacuum packed and stored at -80ºC until analysis.

DESI MSI was carried out using an automated 2D DESI source (Prosolia Inc, Indianapolis, IN, USA) with home-built sprayer assembly mounted to a Q-Exactive FTMS instrument (Thermo Scientific, Bremen, Germany). Analyses were performed at spatial resolution of 65μm in negative ion mode, and mass spectra were collected in the mass range of 80−600 Da with mass resolving power set to 70000 at *m/z* 200 and an S-Lens setting of 100. Methanol/water (95:5 v/v) was used as the electrospray solvent at a flow rate of 1.0 μL/min and a spray voltage of -4.5kV. Distance between DESI sprayer to MS inlet was 7mm, while distance between sprayer tip to sample surface was 1.5mm at an angle of 75°. Nitrogen N4.8 was used as nebulising gas at a pressure of 6.5 bar. Omnispray 2D (Prosolia, Indianapolis, USA) and Xcalibur (Thermo Fisher Scientific Inc) software were used for MS data acquisition. Individual line scans were converted into centroided .mzML format using MSConvert (ProteoWizard toolbox version 3.0.4043) and subsequently into .imzML using imzML converter v1.3. H&E staining was performed post analysis on same tissue sections and the stained sections were imaged at 40x with Aperio CS2 digital pathology scanner (Aperio Tech., Oxford, UK), and visualised with QuPath 0.23 for histopathological annotations performed by the same urogenital pathologist initially responsible for sample collection (AYW). The annotation process involved drawing free-hand regions-of-interest around tumour epithelial glands to minimise signal contamination by surrounding stroma or benign epithelium and ensure adequate comparison with clinical HP-^{13}C-MRI data.

Statistics and reproducibility

Statistical analyses were conducted using GraphPad Prism (version 9.5.1, GraphPad Software, San Diego, CA, USA). Normal distribution of the data was assessed using the D'Agostino-Pearson test (threshold $P \geq 0.05$). A one-tailed Mann-Whitney *U* test was used to assess intergroup differences as our clinically based hypothesis was that BCR-positive lesions would exhibit a more aggressive phenotype reflected by the increase in standard (PSA, ADC) and metabolic ([1-^{13}C]lactate labelling, epithelial MCT1, epithelial *LDHA*, epithelium-to-stroma MCT4 ratio, and DESI MSI-

derived epithelial lactate abundance) biomarkers compared to BCR-negative lesions. *P* values below were 0.05 considered significant. All experiments were independent and standalone.

Data sharing plans

The study dataset will be deposited in Mendeley Data upon acceptance (reserved DOI: 10.17632/cxpc52bwhn.1).

Supplementary references:

1. N. Sushentsev, *et al.*, Hyperpolarised 13C-MRI identifies the emergence of a glycolytic cell population within intermediate-risk human prostate cancer. *Nat. Commun. 2022 131* **13**, 1–12 (2022).
2. N. Sushentsev, *et al.*, The potential of hyperpolarised 13C-MRI to target glycolytic tumour core in prostate cancer. *Eur. Radiol.*, 1–8 (2022).
3. A. Y. Warren, *et al.*, Method for sampling tissue for research which preserves pathological data in radical prostatectomy. *Prostate* **73**, 194–202 (2013).
4. A. Dannhorn, *et al.*, Universal Sample Preparation Unlocking Multimodal Molecular Tissue Imaging. *Anal. Chem.* **92**, 11080–11088 (2020).
